# Adenovirus-mediated delivery of the human IFN-γ gene potentiates the cytotoxicity of daunorubicin against leukemic cells through downregulation of the α4β1 integrin/ILK/apoptosis pathway

**DOI:** 10.3892/ol.2013.1749

**Published:** 2013-12-10

**Authors:** JING ZHANG, HUA WANG, LIANG WANG, WEI-DA WANG, QI-RONG GENG, YUE LU

**Affiliations:** 1 State Key Laboratory of Oncology in South China, Guangzhou, Guangdong 510060, P.R. China; 2 Department of Hematologic Oncology, Sun Yat-Sen University Cancer Center, Guangzhou, Guangdong 510060, P.R. China; 3 Institute of Hematology of Sun Yat-Sen University, Guangzhou, Guangdong 510060, P.R. China

**Keywords:** adenovirus, acute myeloid leukemia, α4β1 integrin, IFN-γ, daunorubicin

## Abstract

The recurrence of acute myeloid leukemia (AML) is primarily attributed to drug resistance and minimal residual disease. In addition, adhesion of hematopoietic tumor cells to bone marrow extracellular matrix via β1 integrins (α4β1 and α5β1) is crucial in this process. In the current study, the viability and antiapoptotic ability of U937 cells exposed to daunorubicin (DNR) were shown to be enhanced when cocultured with the mesenchymal stem cells (MSCs) or MSCs transduced with a recombinant adeno-LacZ vector (MSCs-LacZ), followed by upregulation of the adhesion rate of leukemic cells. Notably, cell viability, antiapoptotic and adhesive ability were reversed when U937 cells were cocultured with the MSCs transduced with a recombinant adeno-IFN-γ vector (MSCs-IFN-γ). Transwell assay showed that cell-cell contact is essential for the protective effects of unmodified MSC and the antitumor effects of IFN-γ-expressing MSCs. Western blot analysis and caspase activity assay results indicated that the α4β1 integrin/ILK/apoptosis pathway contributes to the combination effects of DNR and MSCs-IFN-γ, which was further confirmed by the results of the α4β1 integrin siRNA experiments. Thus, gene-modified MSCs expressing IFN-γ may enhance the cytotoxicity of DNR against leukemic cells through downregulation of the α4β1 pathway and may present a novel promising therapeutic strategy for AML.

## Introduction

Current treatments based on chemotherapy alone for acute myeloid leukemia (AML) cure 30–40% of patients <60 years old and ~10% of patients >60 years old (1). Although complete remission rates for acute leukemia have significantly increased during the past 30 years, the vast majority of patients eventually relapse due to residual disease in the bone marrow (BM). However, the treatments for relapsing AML exhibit high therapeutic effects with high toxicity levels or low therapeutic effects with low toxicity levels, which results in a poor overall outcome for AML. Therefore, the development of new therapeutic strategies is required.

IFN-γ demonstrates marked biological activity associated with antiviral, antibacterial and antitumor mechanisms functioning via the innate and adaptive immune responses (2). Ad-IFN-γ has exhibited significant clinical effects in a variety of malignant tumors, such as cutaneous lymphoma and melanoma (3,4). We have previously reported that mesenchymal stem cells (MSCs)-IFN-γ inhibit the proliferation of chronic myeloid leukemic cells *in vitro*(5). Nevertheless, the correlation between Ad-IFN-γ and AML remains unclear.

Increasing evidence has demonstrated that the adhesion of hematopoietic tumor cells to fibronectin (FN) of the extracellular matrix via β1 integrins confers a multidrug resistance phenotype (6). ILK has been proposed to play a critical role in integrin-mediated signaling, and is capable of interacting with cytoplasmic domains of the integrin β1 subunit and regulating the phosphorylation of AktSer473 (7–9). Previously, Tabe *et al* indicated that ILK/Akt are involved in a proximal signaling pathway critical for the survival of leukemic cells within the BM microenvironment (10). Moreover, it has also been reported that exposure of human fibroblasts to IFN-γ induces a subcortical actin assembly and subsequently reduces the affinity activity of β1 integrin during its engagement with collagen (11). These results have revealed that there may be a correlation between IFN-γ and β1 integrin. Therefore, we hypothesized that IFN-γ reduces the adhesion of acute leukemic cells to MSCs, thereby reversing drug resistance and improving the efficacy of chemotherapy drugs by inhibiting the β1 integrin/ILK/apoptosis pathway. For this purpose, an Ad-IFN-γ vector was constructed and subsequently transduced into human MSCs. Its function was then analyzed in a human AML cell line. MSCs were utilized for the present study since exogenously administered MSCs preferentially engraft at the tumor sites and contribute to the population of stromal fibroblasts. This may allow for the development of a therapeutic strategy based on the local production of biological agents in tumors by gene-manipulated MSCs (12).

## Materials and methods

### Antibodies and reagents

Daunorubicin (DNR) was obtained from Pharmacia and Upjohn, Inc. (Bridgewater, NJ, USA) and the mouse anti-human primary antibodies, β1 integrin, ILK, t-Akt and p-Akt, were purchased from Santa Cruz Biotechnology Inc. (Santa Cruz, CA, USA). The α4β1, α5β1, Bax, Bcl-2, cytochrome *c* and caspase-9, -8 and -3 antibodies, as well as horseradish peroxidase-conjugated secondary antibody were obtained from Cell Signaling Technology, Inc. (Beverly, MA, USA).

### Cell line

The U937 cell line was obtained from the State Key Laboratory of Oncology in South China, Sun Yat-Sen University Cancer Center (Guangzhou, China; purchased from the American Type Culture Collection, Manassas, VA, USA). The cells were maintained in RPMI -1640 medium (Gibco-BRL, Carlsbad, CA, USA) supplemented with 10% fetal bovine serum (FBS), 100 U/ml penicillin and 100 U/ml streptomycin at 37°C in a humidified incubator with a 5% CO_2_ atmosphere and subcultured every 2 days. The cells were demonstrated to be free of mycoplasma.

### Isolation and culture of human leukemic MSCs

Heparinized BM samples were obtained from six patients with AML admitted to the Department of Hematologic Oncology, Sun Yat-Sen University Cancer Center (Guangzhou, China) between October and December 2011. Informed written consent was obtained according to the institutional guidelines under the protocol approved by the Ethical Committee. Human MSCs were isolated and cultured as described previously (13). Primary MSCs were subcultured once cells were considered 80–90% confluent. The subculture time point was every 2–3 weeks and at passages two or three, the MSCs were used for further experiments.

### Immunophenotyping of cultured MSCs

Trypsinized MSCs (2×10^5^) were washed with fluorescence-activated cell sorting (FACS) buffer [2% FBS and 0.1% NaN_3_ in phosphate-buffered saline (PBS)], incubated on ice for 30 min and stained with fluorescein isothiocyanate (FITC)-conjugated mouse monoclonal antibodies (anti-CD34, -CD14 and -CD45; Becton-Dickinson, San Jose, CA, USA) and phycoerythrin-conjugated mouse monoclonal antibodies (anti-CD73, -CD105, -CD90, -CD19 and -HLA-DR; Abcam, Cambridge, UK). Following washing twice with FACS buffer, the cells were fixed with 1% paraformaldehyde. The labeled cells were analyzed using a FACSCalibur (BD Biosciences, Franklin Lakes, NJ, USA) by collecting ≥10,000 cells. The data were analyzed using FlowJo software (Tree Star Inc., Ashland, OR, USA).

### Generation of recombinant adenovirus

The recombinant Ad-IFN-γ and Ad-LacZ vectors were obtained from Sun Yat-Sen University Cancer Center. The constructs were based on the method developed by Mizuguchi and Kay (14) and performed as described previously (15). Briefly, the E1-deleted adenovirus was used to produce non-replicating recombinant adenoviruses, Ad-IFN-γ and Ad-LacZ. The cDNA for human IFN-γ or LacZ was inserted into the E1 region of the adenovirus and transgenic expression was driven by the cytomegalovirus promoter. The adenovirus titer used was 5×10^11^ pfu/ml, as assessed using the TCID50 method.

### Transduction of human MSCs with recombinant adenoviruses

MSCs were plated at a density of 1×10^4^ cells/well in a 96-well plate with each well containing 200 μl medium. Cells were allowed to grow for 24 h prior to the removal of supernatants. The adherent MSCs were washed twice with PBS followed by the addition of serum-free culture medium. The serum-free culture medium was removed following 30 min and the MSCs were then incubated with Ad-IFN-γ or Ad-LacZ at a multiplicity of infection (MOI) of 50, at 37°C in 5% CO_2_ for 1 h. Next, the transduced MSCs (MSCs-IFN-γ or MSCs-LacZ) were cultured in fresh complete culture medium containing 10% FBS, 100 U/ml penicillin and 100 U/ml streptomycin and were used in subsequent experiments.

### Detection of IFN-γ mRNA expression via reverse transcription polymerase chain reaction (RT-PCR)

Total RNA was isolated from MSCs-IFN-γ or MSCs-LacZ using TRIzol Reagent (Invitrogen Life Technologies, Carlsbad, CA, USA) according to the manufacturer’s instructions. First-strand cDNA was synthesized from 1 μg total RNA in a 20-μl reaction mixture using a cDNA synthesis kit (MBI Fermentas Inc., Burlington, ON, Canada). PCR was then performed using the following specific primer pairs: Sense, 5′-ATGAAATATACAAGTTATATC-3′ and antisense, 5′-GTCGAAGAGCATCCCAGTAA-3′ for IFN-γ. The cycling parameters used for all PCR reactions were as follows: One cycle of 96°C for 3 min, 56°C for 1 min and 72°C for 2 min, followed by 24 cycles of 95°C for 1 min, 55°C for 1 min and 72 °C for 1 min. An extension reaction at 72°C for 10 min followed the final cycle. All amplification reactions were performed in a thermal cycler (Biotra, Goettingen, Germany).

### Activity assay of IFN-γ released by transduced MSCs

MSCs were infected with Ad-IFN-γ or Ad-LacZ at a MOI of 50 for 1 h and incubated at 37°C in 5% CO_2_ in complete medium. Following infection, the supernatant of the cultured cells was harvested at 24, 48, 72 and 96 h. An IFN-γ ELISA kit (R&D systems, Minneapolis, MN, USA) was used to measure the human IFN-γ levels according to the manufacturer’s instructions.

### Cell Counting kit-8 (CCK-8) cytotoxicity assay

Cell viability was assessed using CCK-8 (Dojindo Laboratories, Kumamoto, Japan) as described previously (16). In total, 2×10^4^ U937 cells (100 μl) were seeded on non-coated 96-well plates or plates coated with MSCs, MSCs-LacZ and MSCs-IFN-γ. Following an additional 24-h culture or co-culture, U937 cells were exposed to DNR for 24, 48 and 72 h. At the endpoint of the treatments, 10 μl CCK-8 solution was added to each well followed by a 4-h incubation at 37°C. Next, the OD value for each well was read at a wavelength of 450 nm to determine cell viability using a microplate reader (Multiskan; Thermo Fisher Scientific, Waltham, MA, USA). The wells containing only medium and drug (coated with or without MSCs) were used as a control.

For efficacy experiments with Transwell plates, U937 cells (2×10^5^ cells/ml) were plated in the upper well of 24-mm tissue culture Transwell plates on porous inserts (12 μM) and MSCs, MSCs-LacZ or MSCs-IFN-γ were grown in a monolayer in the lower well of the Transwell plates. Following a 24-h coculture, the U937 cells were exposed to DNR for the indicated periods and cell viability was then measured using the previously described method.

### Apoptosis analysis

The Annexin V-FITC/PI apoptosis detection kit (BD Biosciences) was used according to the manufacturer’s instructions. In total, ≥10,000 cells were measured using a FACScan machine (Becton-Dickinson) and the data were analyzed using FlowJo software (Tree Star Inc.). Cells positive for early and late apoptosis markers were combined.

Caspase activity was assessed using caspase colorimetric protease assay kits (Nanjing Keygen Biotech. Co. Ltd., Nanjing, China) for the analysis of the activity levels of caspase-9, -8 and -3 following 48 h of incubation, as described previously (17). Each sample was read at 405 nm using a Genious microtiter plate reader (Tecan Group Ltd., Männendorf, Switzerland).

### Adhesion washing assay

The adhesion washing assay was performed as described previously (18). U937 cells were pre-incubated for 24, 48 and 72 h with DNR prior to attachment. Subsequently, 3×10^4^ cells/well (100 μl) were allowed to adhere to MSC-coated 96-well plates for 3 h. Next, the removal of unattached and weakly attached U937 cells was performed by removing the supernatant followed by two washes with PBS. Adherent cells were incubated with 10 μl CCK-8 solution for 4 h and the plates were then read at 450 nm using a microplate reader (Multiskan; Thermo Fisher Scientific). The unwashed wells were also incubated with CCK-8 and read as total cell optical density (OD). The OD of the MSC-coated well without U937 cells was used as a blank control. Consequently, the percentage of adherent U937 cells was calculated as follows: Adherent U937 cells (%)= (attached cell OD − MSC OD)/(total cell OD − MSC OD ) × 100.

### Western blot analysis

Following 48 h of incubation, western blot analysis was performed as described previously (19). Total and cytoplasmic fractions from U937 cells were prepared using RIPA buffer and a NE-PER Nuclear and Cytoplasmic Extraction reagent kit (Pierce Biotechnology, Inc., Rockford, IL, USA). LumiGLO chemiluminescent reagents (Cell Signaling Technology, Inc.) were used to analyze protein expression and β-actin was used as a loading control.

### Transfection of U937 cells with siRNA

The siRNA against the human integrin subunit α4 (ON-TARGET plus SMARTpool L-005189-00-0005), as well as non-targeting control siRNA (ON-TARGET plus siCONTROL non-targeting pool D-001810-10-05) were obtained from Dharmacon, Inc. (Lafayette, CO, USA). Human U937 cells were transfected using the Lipofectamine 2000 reagent (Invitrogen Life Technologies) according to the manufacturer’s instructions and the transfected cells were used for experiments 2 days later as abovementioned.

### Statistical analysis

Simple descriptive statistics were compared using Student’s t-test when appropriate. Data analysis was performed using SPSS software, version 13.0 (SPSS, Inc., Chicago, IL, USA). All tests were two-tailed and P<0.05 was considered to indicate a statistically significant difference.

## Results

### Immunophenotype of human MSCs

**Figure 1:**
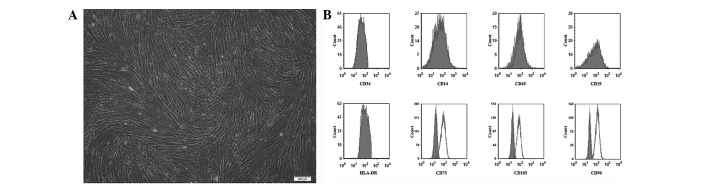
Morphological appearance and immunophenotypes of MSCs. (A) Primary human MSCs (magnification, ×100). (B) Positive and negative surface biomarkers on primary MSCs were analyzed by FACSCalibur. The black curves present tested cells and the shadow areas present isotype controls. MSCs, mesenchymal stem cells; FACS, fluorescence-activated cell sorting.

The primary MSCs exhibited similar spindle-shaped and fibroblastic morphological characteristics and were 80–90% confluent within 2–3 weeks (Fig. 1A). The MSCs expressed typical positive surface biomarkers (CD73, CD105 and CD90) and negative biomarkers (CD34, CD45, CD14, CD19 and HLA-DR) (Fig. 1B).

### Detection of IFN-γ mRNA and protein of MSCs-IFN-γ

**Figure 2:**
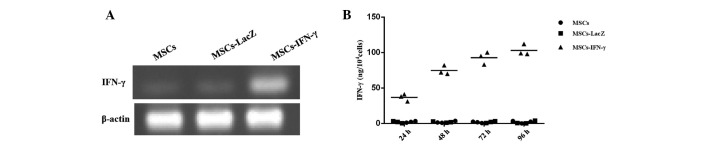
Expression of IFN-γ in MSCs transduced with the recombinant adenoviruses. (A) RT-PCR showed that the expression levels of IFN-γ mRNA were significantly higher in MSCs-IFN-γ than in the two control groups. (B) IFN-γ protein levels were evidently elevated in MSCs-IFN-γ, while its expression was fairly low in the two control groups. Experiments were repeated three times and results are presented as the mean ± SD. MSCs, mesenchymal stem cells; RT-PCR, reverse transcription polymerase chain reaction.

To verify transduction efficacy, RT-PCR was used to detect IFN-γ mRNA following 24 h of transduction. An MOI of 50 was selected since it had been previously found to yield a high transduction efficiency without clear cytopathic effects. The expression levels of IFN-γ mRNA were significantly higher in MSCs-IFN-γ than in MSCs or MSCs-LacZ (Fig. 2A). Additionally, IFN-γ protein expression levels were elevated in MSCs-IFN-γ at various time points, which tended to be positively associated with time following 96 h of transduction, peaking at 103±7.81 ng/10^4^ cells. However, the expression was fairly low (<4 ng/10^4^ cells) in the two control groups (Fig. 2B).

### MSCs-IFN-γ enhances DNR-induced cytotoxicity

**Figure 3:**
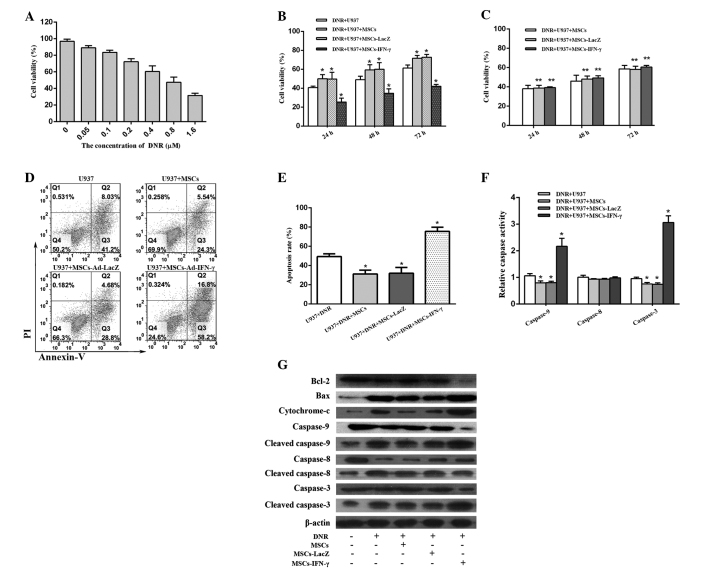
CCK-8 cytotoxicity assay and apoptosis analysis. (A) U937 cells were incubated with different concentrations of DNR alone for 48 h. (B) U937 cells were incubated with DNR alone or with a combination of DNR and MSCs or MSCs transduced with recombinant adenoviruses for 24, 48 and 72 h. (C) Transwell experiments revealed that U937 cell viabilities were not significantly different among the three cocultured groups at different time points. P, no significance. (D) Cytograms show the representative apoptotic results following the 48-h incubation. (E) Graph shows the percentage of apoptotic cells. (F) Caspase activity assay was performed in U937 cells (with different treatments) following a 48-h incubation. (G) U937 cells were incubated in various conditions for 48 h, then the key proteins associated with the apoptosis were analyzed by the immunoblot. Experiments were repeated three times and results are presented as the mean ± SD. ^*^P<0.05, vs. the U937 group incubated with DNR alone; ^**^P>0.05, vs. the U937 group incubated with DNR and MSCs. CCK-8, Cell Counting kit-8; DNR, daunorubicin; MSCs, mesenchymal stem cells.

To confirm the suitable dosage of DNR to be used in the present study, the IC_50_ value of DNR was first identified for U937 cells. Following a 48-h incubation period, the IC_50_ value of DNR in U937 cells was 0.655±0.087 μM (Fig. 3A); therefore, 0.655 μM DNR was used for each of the following experiments. U937 cells incubated with MSCs or MSCs-LacZ were found to exhibit significantly enhanced survival compared with those incubated without MSCs, while U937 cells incubated with MSCs-IFN-γ grew significantly slower than the other three groups. At 48 h of incubation, the cell viability of U937 cells was 34.67±2.67% for the group transduced with Ad-IFN-γ, 50.00±2.08% for the uncoated group, 59.33±3.18% for the group coated with MSCs and 60.00±4.04% for the group transduced with Ad-LacZ. Furthermore, similar results in cell viability were observed at 24 and 72 h of incubation among the different groups (Fig. 3B). These results implied that the U937 cell-stromal cell interaction contributes to the enhanced survival of AML cells treated with DNR. Notably, following the transduction of MSCs with Ad-IFN-γ, the survival advantage of U937 cells was reversed. By avoiding cell-cell contact through the use of Transwells, the protective effects of unmodified MSCs and the antitumor effects of IFN-γ-expressing MSCs were lost (Fig. 3C), suggesting a requirement for cell-cell contact.

### MSCs-IFN-γ potentiates the antileukemic effect of DNR via apoptotic mechanisms

Notably, following the 48-h incubation, the apoptotic rate for the group transduced with Ad-IFN-γ (75.56±4.29%) was statistically higher than that of the group transduced with Ad-LacZ (31.96±5.99%), the non-transduced group (31.22±2.32%) and the non-coated group (49.25±1.89%) (Fig. 3D–E). These results clearly exhibited that the pro-apoptosis mechanism is predominantly responsible for the enhancement of the antileukemic effects of DNR imposed by MSCs-IFN-γ. However, following a 72-h incubation, the apoptotic rate in the group transduced with Ad-IFN-γ was slightly higher than that in the other three groups and the differences were not statistically significant (data not shown). The reason for this observation remains unclear, although, it is partly attributable to an increase in viable cells for an extension of incubation time.

To further confirm the changes in caspase levels observed via the Annexin V/PI assay, a caspase colorimetric protease assay was performed to evaluate the activity levels of caspase-9, -8 and -3. The caspase-8 activity levels were found to exhibit no distinct differences among the various groups, while the activity levels of caspase-9 and -3 were statistically reduced when U937 cells were adhered to MSCs or MSCs-LacZ and increased when the cells were adhered to MSCs-IFN-γ (Fig. 3F).

### MSCs-IFN-γ reduces the adhesion ability of U937 cells

**Figure 4:**
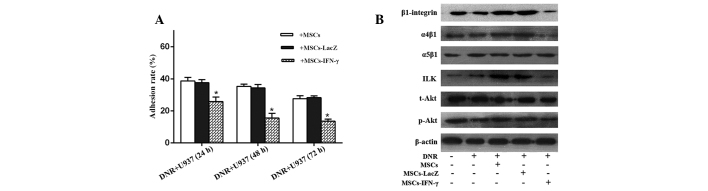
Adhesion rate assay and associated adhesive molecules. (A) Adhesion washing assay showed that the adhesion rate in the group transduced with Ad-IFN-γ was significantly lower than that in the groups transduced with or without Ad-LacZ following pre-incubations of 24, 48 and 72 h. (B) U937 cells were incubated in various conditions for 48 h, then the vital proteins associated with the adhesion were analyzed by immunoblot. Experiments were repeated three times and results are presented as the mean ± SD. ^*^P<0.05, vs. the two groups containing MSCs. MSCs, mesenchymal stem cells; DNR, daunorubicin.

Based on the abovementioned cell viability results, we hypothesized that a correlation exists between the antileukemic effect of IFN-γ released by gene-manipulated MSCs and U937 cell adhesion. With the extension of pre-incubation time, the adhesion rates for the three coated groups appeared to be reduced. However, the adhesion rates significantly declined considering that pre-incubated U937 cells were cocultured with MSCs-IFN-γ. A decrease was not observed when pre-incubated U937 cells were cocultured with MSCs-LacZ; the adhesion rates were similar to those in the group coated only with MSCs. Following the 48-h pre-incubation, the adhesion rate was 15.57±1.69% for the group transduced with Ad-IFN-γ, while it was 34.33±1.22 and 35.03±0.80% for the group transduced with Ad-LacZ and the non-transduced group, respectively (Fig. 4A).

### Downregulation of the α4β1 integrin/ILK/apoptosis pathway may contribute to the apoptosis of U937 cells incubated with DNR and MSCs-IFN-γ

Following an incubation period of 48 h, cleaved caspase-9 and -3 levels in the group transduced with Ad-IFN-γ were significantly higher than those observed in the uncoated groups or the groups coated with MSCs and MSCs-LacZ. However, caspase-8 activity exhibited no clear differences among all groups, with the exception of the U937 group (Fig. 3G). These results suggest that apoptosis of U937 cells is mediated by the mitochondrial pathway, therefore, cytoplasmic cytochrome *c* levels were also examined. Cytochrome *c* levels in the cytosol were significantly elevated in the group transduced with Ad-IFN-γ and exhibited trends similar to the cleaved caspase-9 and -3 levels in all groups. This further confirmed that the combination effect of DNR and IFN-γ is mediated by the mitochondrial apoptosis pathway.

To further investigate the molecular mechanism behind the phenomenon of U937 cell apoptosis, β1 integrin, α4β1, α5β1, ILK, t-Akt, p-Akt, Bcl-2 and Bax protein expression levels were analyzed. The varying trends of these molecules were found to negatively correlate with those of the cleaved caspases and cytochrome *c* levels for all the DNR-containing groups, with the exception of α5β1 and t-Akt (for which the expression levels remained unchanged) and bax (for which the expression pattern was found to positively correlate with cytosol cytochrome *c* and cleaved caspase levels). The group transduced with Ad-IFN-γ, β1 integrin, α4β1, ILK, p-Akt and Bcl-2 exhibited the lowest expression levels. Conversely, these proteins were highly upregulated in the groups coated with MSCs or MSCs-LacZ (Figs. 3G and 4B), for which the highest levels of cell viability were observed. Furthermore, the expression levels of β1 integrin, α4β1, α5β1 and ILK in the U937 plus DNR group were similar to those observed in the U937 group alone, which suggests that DNR does not affect integrin levels in U937 cells. The immunoblot results were found to correlate with the aforementioned caspase activity assay.

### siRNA-mediated knockdown of the integrin subunit α4 reverses the survival advantage of U937 cells adherent to MSCs-LacZ

**Figure 5:**
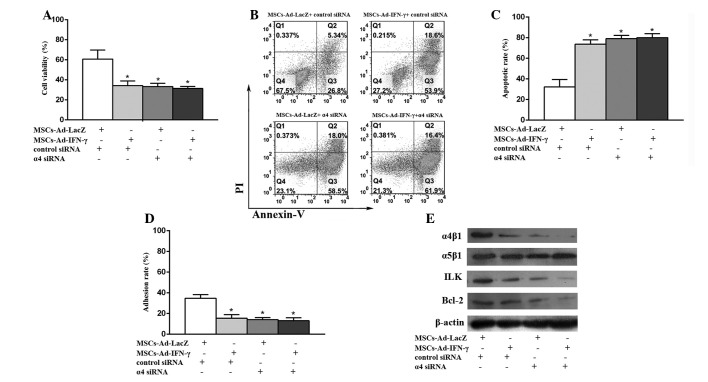
U937 cells incubated with DNR and the recombinant adenoviruses in the presence of α4 or control siRNA for 48 h. (A) Cell viability assay, (B and C) apoptotic analysis, (D) adhesion rate assay and (E) immunoblot analysis of key proteins involved in the α4β1 integrin pathway. Experiments were repeated three times and results are presented as the mean ± SD. ^*^P<0.05, vs. MSCs-Ad-LacZ and control siRNA. DNR, daunorubicin; MSCs, mesenchymal stem cells.

In the group incubated with DNR and MSCs-LacZ, the cell viabilities (Fig. 5A) and adhesion rates (Fig. 5D) were significantly reduced. In addition, the apoptotic rates (Fig. 5B–C) were greatly enhanced and the key proteins (Fig. 5E) in the integrin pathway, such as ILK and Bcl-2, were evidently downregulated with the addition of α4 siRNA, compared with the addition of control siRNA. For the U937 cells incubated with DNR and MSCs-IFN-γ, the cell viabilities and adhesion rates were slightly reduced and apoptotic rates were slightly enhanced in cases of α4 siRNA, compared with the control siRNA. These results confirmed that α4β1 is critical in the adhesion of U937 to MSCs, and that MSCs-IFN-γ promotes the pro-apoptosis effects of DNR in U937 cells via the α4β1 integrin/ILK/apoptosis pathway.

## Discussion

A number of previous studies have observed the phenomenon of cell adhesion mediated drug resistance (CAM-DR) in various hematologic malignancies. Damiano *et al* reported that K562 cells adhered to FN via α5β1 provide significant resistance against apoptosis induced by a number of DNA-damaging agents, including melphalan, mitoxantrone and γ-irradiation (6). Growing AML cells on HS-5 stroma reduces DNR- or cytarabine-induced apoptosis (20). The adhesion of U937 or HL60 cells to FN via β1 integrins inhibits apoptosis induced by a variety of chemotherapy drugs (21,22). Acting as an important component of the BM stroma, MSCs play a vital role in CAM-DR in types of hematological cancer. MSCs are typically devoid of hematopoietic markers (CD45, CD34, CD14 or CD11b, CD79α or CD19 and HLA-DR), but positively express specific stromal cell markers (CD73, CD105 and CD90) (23). In the present study, MSCs were isolated from AML patient BM and the observations were found to be consistent with the abovementioned studies. Specifically, primary MSCs exhibited typical immunotypes and the pro-apoptosis effects of DNR were reduced when U937 cells were adhered to MSCs or MSCs-LacZ *in vitro*. Moreover, the Transwell assay results indirectly suggested that contact is essential for the effects between U937 cells and MSCs, which is likely to be responsible for minimal residual disease (MRD) in patients with AML. Novel strategies that improve AML patient outcome are urgently required, particularly for patients who exhibit a failure of remission induction or relapse, for which chemotherapy resistance is likely to play a major role in poor survival (24).

Although IFN-γ has been previously recommended for a broad range of indications and is used more frequently than before in the clinic, its application remains hindered by the systemic delivery of high dosages to yield an enhanced therapeutic effect. In addition, treatment has been associated with serious adverse drug reactions. Systemic administration is most likely to yield an unequal and unpredictable distribution of IFN-γ, thereby suggesting that the drug concentration in the blood stream does not necessarily reflect the therapeutic result, particularly for MRD. It has been previously shown that regional secretion and limited diffusion of paracrine IFN-γ into the blood stream minimizes drug toxicity and maximizes treatment outcome. In the current study, a recombinant Ad-IFN-γ vector was constructed and transduced into MSCs, thereby, inducing IFN-γ release *in vitro*. Due to their distinct homing ability, MSCs may be useful as delivery agents to target tumors. Therefore, MSCs-IFN-γ may exhibit antileukemic effects *in vivo*. Currently, MSCs have been used as delivery agents for a number of cytokines that inhibit tumor growth. The use of TRAIL-expressing MSCs has been reduced and, in specific cases, eliminated metastatic disease in a previous murine lung metastasis model (25). However, MSCs expressing IFN-α have been found to reduce the proliferation of transformed cells by enhancing apoptosis in a previous melanoma lung metastasis model (26). In addition, Studeny *et al* previously suggested that MSCs with forced expression of IFN-β inhibit the growth of malignant cells *in vivo*. Notably, this effect requires the integration of MSCs in tumors and was not achieved by systemically delivered IFN-β or IFN-β produced by MSCs at a site distant from the tumor (12).

Integrins are heterodimeric receptors consisting of one α and one β subunit. The β1 integrin subfamily is composed of 12 members, as defined by the participating α subunit (α1–α12), which is widely expressed and constitutes a major class of integrins (27). The α4β1 and α5β1 are typically expressed on leukemic cells. In the present study, cell viability, adherent ability and β1 integrin and α4β1 protein levels (not α5β1) were found to enhance when leukemic cells were adhered to MSCs or MSCs-LacZ, while these factors were reduced when U937 cells were adhered to MSCs-IFN-γ. The conclusion was also validated via Annexin V/PI apoptosis and caspase activity assays. Moreover, the protective effect of MSCs-LacZ was lost with the addition of α4 siRNA, which indicates that α4β1 plays a key role in the adhesion of U937 cells to MSCs and that the pro-apoptotic effect of MSCs-IFN-γ is mediated by the downregulation of α4β1. In previous years, controversy has arisen with regard to the importance of leukemic adhesion and cell survival involving α4β1 and α5β1. Matsunaga *et al* demonstrated that the interaction of α4β1 expressed on leukemic cells with stromal FN is crucial in MRD of AML (22). However, in adherent U937 cells, α5β1 but not α4β1 enhanced the resistance to TNFα-induced apoptosis, although extrinsic and intrinsic apoptotic pathways are under the control of α5β1 and GSK3β (28). The reason why only α4β1 or α5β1 play a role in these previous studies remains unclear and is not explained by their distinct expression patterns on the surface of various leukemiac cells, since α4β1 and α5β1 are highly expressed in U937 cells. This phenomenon is partly explained by the observation that α4β1 and α5β1 bind various specific FN domains, which then determines whether effects are likely to occur or not.

ILK is an ankyrin repeat-containing serine-threonine protein kinase that interacts directly with the cytoplasmic domain of the β1 integrin subunit as an essential element in the regulation of integrin signaling. This is modulated by integrin ligation in a PI3K-dependent manner and stimulates the phosphorylation of Akt at Ser473 (10). Of note, the present study showed that the adhesion of α4β1 expressed on U937 cells to MSCs or MSCs-LacZ enhanced ILK/Bcl-2 activity, which led to DNR resistance. MSCs-IFN-γ reduced ILK/Bcl-2 activity and promoted the apoptosis of U937 cells. Moreover, the observations were also found to correlate with a previous study by Matsunaga *et al*, who suggested that the interaction between α4β1 expressed on leukemic blasts and FN on stromal cells activate PI3K/Akt/Bcl-2 signaling, an important determinant of AML chemosensitivity and the level of MRD in AML patients (22). To the best of our knowledge, the current study is the first to report that Ad-IFN-γ enhances the cytotoxicity of DNR against U937 cells via the α4β1/ILK/apoptosis pathway.

In conclusion, gene-modified MSCs expressing IFN-γ may present a novel promising therapeutic strategy for AML. Further investigations are necessary to confirm the observations of the current study in systemic AML xenograft models.
